# Pentavalent rotavirus vaccine effectiveness among children in Shenzhen, China: A population-based test-negative design with directed acyclic graphs bias adjustment

**DOI:** 10.1016/j.imj.2025.100201

**Published:** 2025-09-05

**Authors:** Zian Lin, Weiyi Cai, Yanan Liu, Juan Liu, Hongbiao Chen, Shaojian Xu, Qiuju Xie, Danting Lou, Yuying Zhang, Hairong Nan, Jiahui Li, Lixian Su

**Affiliations:** a Department of Pediatrics, Shenzhen Longhua District Maternity and Child Healthcare Hospital, Shenzhen 518000, Guangdong Province, China; b Department of Central Laboratory, Shenzhen Longhua District Maternity and Child Healthcare Hospital, Shenzhen 518000, Guangdong Province, China; c Department of Epidemiology and Infectious Disease Control, Longhua Key Discipline of Public Health for the Prevention and Control of Infectious Diseases, Shenzhen Longhua Center for Disease Control and Prevention, Shenzhen 518109, Guangdong Province, China; d Department of Laboratory Medicine, Shenzhen Longhua Center for Disease Control and Prevention, Shenzhen 518109, Guangdong Province, China; e Zhangxi Community Health Service Center, Shenzhen Longhua District Center Hospital, Shenzhen 518110, Guangdong Province, China; f Department of Disease Prevention and Control, Shenzhen Bantian Public Health Service Center of Longgang, Shenzhen 518129, Guangdong Province, China; g Department of Endocrinology, Shenzhen Longhua District Maternity and Child Healthcare Hospital, Shenzhen 518000, Guangdong Province, China; h Department of Pediatrics, Shenzhen Futian District Maternal and Child Health Hospital, Shenzhen 518045, Guangdong Province, China

**Keywords:** Pentavalent rotavirus vaccine, Vaccine effectiveness, Rotavirus gastroenteritis, Directed acyclic graph

## Abstract

**Background:**

China's Notifiable Disease Reporting System comprehensively collects data on pediatric acute gastroenteritis cases from all levels of healthcare. This study evaluated the effectiveness of the pentavalent rotavirus vaccine against rotavirus gastroenteritis in children under five years of age during seasonal epidemic periods.

**Methods:**

Using a test-negative design, we analyzed laboratory data from diarrhea patients aged 2–59 months who sought medical care in Longhua District, Shenzhen, Guangdong Province, China between January 2020 and March 2024. Vaccination history and potential confounders, including sex, household registration status, prematurity, and birth weight, were collected retrospectively. Directed acyclic graphs were used to identify adjustment variables. Vaccine effectiveness was calculated as (1 − adjusted odds ratio) × 100% using unconditional logistic regression, comparing rotavirus-positive cases with rotavirus-negative controls.

**Results:**

The incidence of rotavirus gastroenteritis peaked and then progressively declined from January 2020 through March 2023, with no seasonal resurgence observed in the first quarter of 2023. Analysis of 928 epidemic-season cases (674 RV+; 254 RV–) revealed an overall pentavalent rotavirus (RV5) vaccine effectiveness of 79.6% (95% CI: 68.9%–86.6%). Dose-specific vaccine effectiveness varied substantially: 1 dose vaccine effectiveness registered at 26.7% (95% CI: −162.1%–79.5%), rising to 76.6% (95% CI: 46.2%–89.8%) for 2 doses, and reaching 82.7% (95% CI: 72.2%–89.2%) for 3 doses. Age-stratified analysis showed vaccine effectiveness of 79.5% (95% CI: 56.8%–90.3%) in children aged 2–11 months, 83.8% (95% CI: 66.4%–92.2%) for 12–23 months, and 79.6% (95% CI: 68.9%–86.6%) in those aged 24–35 months, while vaccine effectiveness was 31.2% (95% CI: −251.8%–86.5%) in children aged 36–59 months.

**Conclusions:**

A three-dose regimen of pentavalent rotavirus vaccine provides significant effectiveness against rotavirus gastroenteritis for children under 36 months of age during epidemic seasons, thereby alleviating the burden on public health systems.

## Highlights

• RV5 shows 82.7% effectiveness against rotavirus gastroenteritis in real-world epidemic settings.
• DAG-based causal inference first applied in pediatric vaccine studies using China's NDRS data.
• Preterm infants achieve 91.8% protection with full RV5 vaccination, surpassing term infants.
• Strong dose-dependent protection: 76.6% after two doses, 82.7% after three doses.
• Findings support urgent inclusion of RV5 into national immunization programs.

## 1 Introduction

Rotavirus (RV) infection remains a leading global cause of pediatric hospitalization, severe morbidity, and mortality due to acute gastroenteritis (AGE), particularly in low-income countries.^1^ Although the introduction of rotavirus vaccination has significantly reduced the incidence of rotavirus gastroenteritis (RVGE)-related hospitalizations and deaths in the post-vaccine era, rotavirus continues to be the second most common pathogen (after norovirus) identified in outpatient diarrheal cases.^2^

Rotavirus vaccination is a globally recognized and highly effective public health intervention for preventing RVGE.^3^ Despite the substantial disease burden in China, rotavirus vaccines have not yet been incorporated into the National Immunization Program and must be paid for out-of-pocket by parents.^4^ Families must choose among three available market options: the Lanzhou lamb rotavirus vaccine (LLR; Lanzhou Institute of Biological Products, Lanzhou, Gansu Province, China; ≈ ¥174/$23.93 per dose), the pentavalent rotavirus vaccine (RV5; Merck Sharp & Dohme, New Jersey, USA; ≈ ¥314/$43.19 per dose), or the trivalent human–sheep reassortant vaccine (LLR3; Lanzhou Institute of Biological Products, Lanzhou, Gansu Province, China; ≈ ¥254/$34.94 per dose). Historical data indicate that reductions in RVGE hospitalizations^5^ and mortality^6^ observed before 2018 were partly attributable to improvements in medical care.^7^ Since the introduction of RV5 in 2018, studies conducted in Beijing,^8^ Shanghai,^9^^,^^10^ and Guangdong Province^11^ have consistently demonstrated its high effectiveness against severe RVGE. Nevertheless, vaccination coverage remains critically low—with reported rates of 22.4 % in Beijing^12^ and 29.8 % in Shanghai^13^—resulting in a persistent disease burden among children under five years of age.

To address public health challenges posed by infectious diseases such as AGE, China implemented the Infectious Disease Prevention and Control Law of China (enacted in 1989, revised in 2020),^14^ establishing a comprehensive legal framework for disease control. This law mandates that medical institutions report newly diagnosed cases of infectious diarrhea through the national surveillance system, adhering to tiered reporting timelines: Class A diseases (e.g., cholera) must be reported within 2 hours of diagnosis, while Class B (e.g., bacillary/amoebic dysentery, typhoid/paratyphoid) and Class C diseases (including other infectious diarrheas) must be reported within 24 hours. Each report must include essential patient information, precise symptom onset time, credentials of the reporting facility, and definitive laboratory results.

Operated by the Chinese Center for Disease Control and Prevention, the Notifiable Disease Reporting System (NDRS) serves as the core platform for legally mandated infectious disease surveillance.^15^ Since 2004, the NDRS has enabled real-time direct reporting from all levels of healthcare facilities across the country, allowing for dynamic monitoring of infectious diseases, including diarrheal illnesses.^16^ As China's most extensive national surveillance infrastructure, the NDRS provides a robust foundation for real-world vaccine effectiveness (VE) research. However, studies utilizing this system to evaluate rotavirus vaccine protection remain limited.^17^

Shenzhen, a megacity in southern China with over 20 million residents, maintains a robust infectious disease surveillance system well-suited to address this evidence gap. The city comprises nine districts with more than 890 community health centers and 159 general hospitals. Approximately 95 % of residents have electronic health records,^18^ and all medical institutions are legally required to report initial infectious diarrhea diagnoses within the mandated timeframes. According to the NDRS and a comprehensive analysis of rotavirus genotype trends in China (2020–2024), the rotavirus strains circulating in Guangdong Province are predominantly G9P[8], followed by reassortant strains such as G8P[8].^19^ Although the G9 type is not directly covered by the RV5 vaccine (which contains G1–G4), evidence suggests that its pentavalent human–bovine reassortant design elicits broad immune responses targeting conserved epitopes of VP4 and VP7, resulting in highly effective cross-protection against G9 strains.^20^

This study systematically evaluates the protective effectiveness of the RV5 vaccine against RVGE in children during epidemic seasons, using NDRS data from Longhua District, Shenzhen, Guangdong Province, China.

## 2 Materials and methods

### 2.1 Study design and data sources

RV prevalence is significantly influenced by meteorological factors^21^ and vaccination coverage,^5^ with RVGE epidemic seasons in southern China typically occurring from December to March.^4^ This study employed a test-negative design—a modified case-control approach that reduces healthcare-seeking behavior bias and is widely used for real-world VE evaluations.^22^ We analyzed laboratory-confirmed infectious diarrhea cases extracted from the NDRS. The effectiveness of RV5 was assessed by comparing RV-positive cases with RV-negative controls during epidemic seasons. Analyses were conducted to evaluate dose-specific protection (1, 2, and 3 doses) and were stratified by age group (2–11 months, 12–23 months, 24–35 months, and 36–59 months) to elucidate age-specific immune protection. To minimize seasonal confounding, only cases occurring between January and March were included.

This retrospective observational study utilized de-identified data from the infectious disease reporting system. It involved no new sample collection, data acquisition, or patient intervention. All research data were kept strictly confidential. The study met the criteria for a waiver of informed consent as per the Declaration of Helsinki and the Council for International Organizations of Medical Sciences guidelines (https://cioms.ch/wp-content/uploads/2017/01/WEB-CIOMS-EthicalGuidelines.pdf). The study protocol was approved by the Ethics Committee of Shenzhen Longhua District Maternity and Child Healthcare Hospital (Approval No.: SRE-TCFR/2024012), which included a waiver of informed consent.

### 2.2 Data collection and participants

**Fig. 1:**
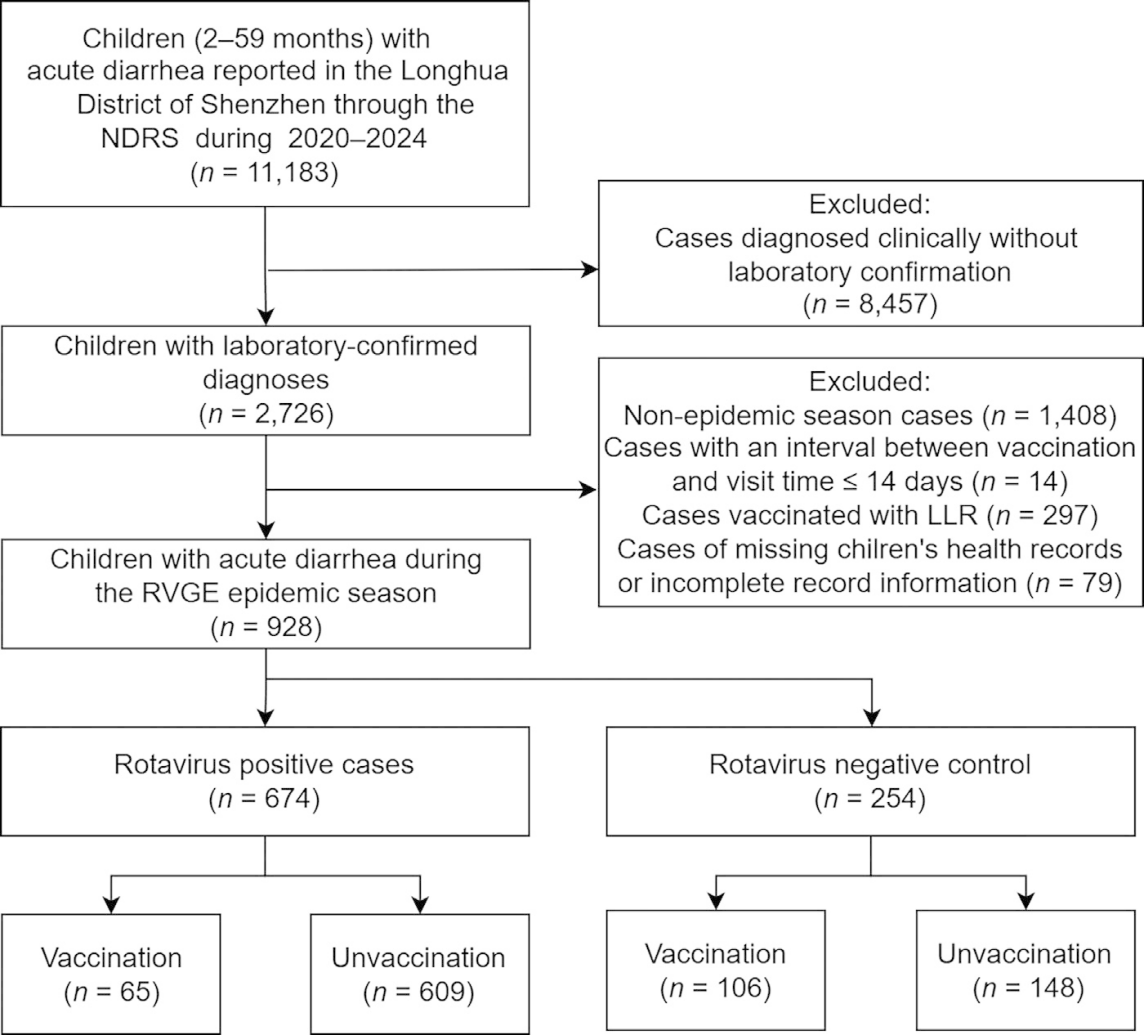
Flow diagram showing the distribution of rotavirus-positive cases and rotavirus-negative controls by vaccination status from January 2020 to March 2024. *Abbreviations*: NDRS, Notifiable Disease Reporting System; LLR, Lanzhou lamb rotavirus vaccine; RVGE, rotavirus gastroenteritis.

Demographic and etiological data were retrieved from the NDRS and the Shenzhen Maternal and Child Health Management System. RV5 vaccination histories were obtained from the Shenzhen Immunization Program Information Management System. The NDRS aggregated cases based on residential address to account for cross-provincial healthcare utilization within the study area (Fig. 1).

#### Inclusion criteria

(1) Children born after October 2019, aged 2–59 months, diagnosed with acute gastroenteritis and eligible for RV5 vaccination;
(2) Residence in Longhua District, Shenzhen;
(3) Laboratory-confirmed infectious diarrhea;
(4) First detection of RV-positive status or any RV-negative result;
(5) Complete demographic, symptom onset, and laboratory data;
(6) Verifiable vaccination records.

#### Exclusion criteria

(1) Birth before October 2019 (ineligible for RV5 vaccination);
(2) Non-residence in Longhua District;
(3) Previous vaccination with LLR;
(4) Administration of RV5 ≤ 14 days before the onset of AGE;
(5) Missing critical data;
(6) Unavailable vaccination records;
(7) Repeat RVGE infections.

### 2.3 Assays and study endpoints

During epidemic seasons, rotavirus replicates actively with high viral loads (mean: 1.99 × 10⁷ copies/g)^23^ and is shed early in infection. The colloidal gold immunoassay detection threshold for RV is 1 × 10³ copies/g,^24^ with a sensitivity of 84.4 % (95 % confidence interval [CI]: 83.6 %–84.8 %) and specificity of 100.0 % (95 % CI: 98.5 %–100.0 %) compared to RT-PCR.^25^ Its simplicity and low cost—requiring no cold chain—make it suitable for rapid testing for rotavirus, adenovirus, and norovirus in pediatric and emergency settings.^26^ Cases occurring outside the epidemic season were excluded due to potentially shorter and more variable viral shedding windows, lower viral loads, and reduced test accuracy. Although reagent details were not recorded in the NDRS, all hospitals procured standardized test kits through government bidding platforms.

### 2.4 Case and control definitions

Case Group (RVGE+): Laboratory-confirmed rotavirus-positive cases from the NDRS (including weak positives), regardless of co-detection of non-target pathogens.

Control Group (RVGE–): Laboratory-confirmed rotavirus-negative cases from the NDRS that tested positive for other non-target viral or bacterial pathogens.

Only the first reported episode per child was included. Repeat infections in subsequent seasons were excluded to avoid bias from naturally acquired immunity. Pathogen distribution details are provided in Supplementary Table S1.

### 2.5 Vaccination exposure definition

Given rotavirus's incubation period of 1–3 days and peak shedding at 4–7 days post-infection,^27^ as well as potential vaccine-strain shedding for up to 15 days after RV5 vaccination,^28^ “effective vaccination” was defined as administration of RV5 dose(s) more than 15 days before the onset of AGE. According to manufacturer guidelines, the first dose of RV5 must be administered by 6 weeks of age (≤ 42 days), and the series should be completed by 8 months of age.

Note: The LLR vaccine, which begins at 2 months of age and requires annual boosting, was excluded from this study. While its real-world effectiveness has been established,^17^ the current analysis focuses exclusively on RV5.

### 2.6 Covariates and directed acyclic graph (DAG) construction

Potential confounders were systematically evaluated across four domains. First, individual factors: sex, age at onset, gestational age (preterm/term),^29^ birth weight (term low birth weight: < 2500 g, preterm low birth weight: < 2500 g, normal weight: 2500–3999 g, or overweight: ≥ 4000 g),^30^ and neonatal health status (normal, malnourished, overweight, or high-risk hospitalization).^31^ These may influence vaccination eligibility (e.g., missed 42-day window), parental willingness,^32^^,^^33^ and susceptibility to RVGE due to immune immaturity mechanisms such as impaired antigen presentation or low mucosal IgA.^29^, ^30^, ^31^ Second, biological factors: breastfeeding patterns (exclusive, mixed, or formula feeding during 0–6 months). Breast milk antibodies may attenuate vaccine effectiveness,^34^ while non-breastfed infants may face higher risks of severe disease.^35^ Third, seasonal and temporal factors: epidemic intensity fluctuations,^36^^,^^37^ cold-weather impacts on health behaviors,^38^ onset year (2020–2024), month (January–March), and effects of population mobility during Chinese New Year.^36^ Finally, socioeconomic proxies: household registration (Shenzhen local vs. non-local) as an indicator of health insurance access affecting vaccination uptake^39^ and healthcare-seeking behavior;^40^ residence type (commercial housing, urban village, or factory dormitory) as a composite proxy for parental economic status (high/moderate/low) and education level (advanced/secondary/low),^41^ which may affect living conditions and disease risk;^42^^,^^43^ and residential area (administrative street) reflecting disparities in healthcare access and population density.^42^^,^^44^

**Fig. 2:**
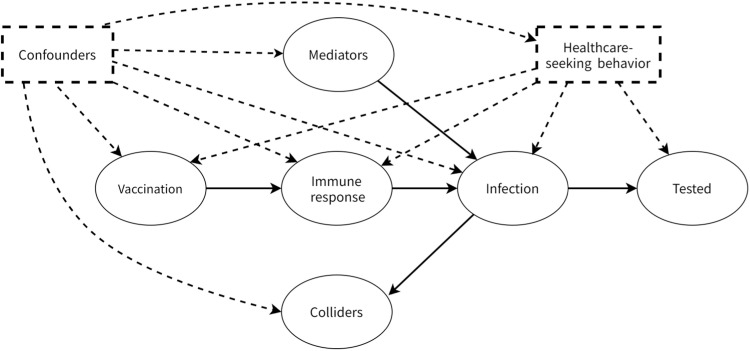
Simplified DAG illustrating causal pathways between RV5 vaccination, potential confounders, and laboratory-confirmed rotavirus diarrhea cases, including bias control adjustment strategies. *Notes:* Health-seeking behavior is adjusted for via the test-negative design. Solid arrows: Causal pathways (cause → effect). Dashed lines: Controlled bias pathways. Dashed-line squares: Adjustment set (age, household registration, prematurity, birth weight, neonatal health status, breastfeeding, residence area/type, season/year/month of onset, health-seeking behavior). Mediators: Feeding habits, lifestyle. Colliders: Post-onset outcomes. *Abbreviations*: DAG, directed acyclic graph; RV5, pentavalent rotavirus.

All covariates represented pre-exposure factors not on the causal pathway between vaccination and outcome. Using DAG to visualize confounding pathways,^45^^,^^46^ a minimal sufficient adjustment set was constructed to control for confounding bias, and mediators (e.g., feeding habits) and colliders (e.g., body mass index after disease onset) were excluded from the adjustment set to avoid introducing additional bias.^47^ Measured variables are detailed in Table 1; unmeasured variables included parental education, lifestyle, and post-onset clinical parameters (Fig. 2). The complete DAG topology and model scope are provided in supplementary materials (Fig. S1). Interactive validation of causal pathways is available via the DAGitty platform (https://www.dagitty.net/dags.html).

**Table 1:** Demographic characteristics of patients with infectious diarrhea pathogens.

Characteristics	Cases, *n* (%) (*n* = 674)	Controls, *n* (%) (*n* = 254)	*χ*^2^	*p*
Sex			1.8	0.181
Male	399 (59.2)	138 (54.3)		
Female	275 (40.8)	116 (45.7)		
Gestational age			10.5	0.001
Term	611 (90.7)	211 (83.1)		
Preterm birth	63 (9.3)	43 (16.9)		
Birth weight			4.2	0.243
Normal	617 (91.5)	232 (91.3)		
Non-preterm low weight	4 (0.6)	2 (0.8)		
Low birth weight preterm	18 (2.7)	12 (4.7)		
Overweight	35 (5.2)	8 (3.1)		
Health status at one-month check-up			8.2	0.043
Normal	465 (69.0)	150 (59.1)		
Malnourished	114 (16.9)	56 (22.0)		
Obesity	65 (9.6)	33 (13.0)		
Other high-risk children	30 (4.5)	15 (5.9)		
Breastfeeding at 6 months of age			5.6	0.06
Exclusive breast milk	115 (17.1)	30 (11.8)		
Blend	95 (14.1)	47 (18.5)		
Artificial	464 (68.8)	177 (69.7)		
Household registration			0.8	0.377
Shenzhen local registration	252 (37.4)	103 (40.6)		
Non-local registration	422 (62.6)	151 (59.4)		
Sub-district			47.3	< 0.001
Longhua	160 (23.7)	75 (29.5)		
Dalang	128 (19.0)	47 (18.5)		
Minzhi	168 (24.9)	79 (31.1)		
Guanlan	99 (14.7)	17 (6.7)		
Guanhu	56 (8.3)	9 (3.5)		
Fucheng	62 (9.2)	16 (6.3)		
Other	1 (0.1)	11 (4.3)		
Residential status			0.13	0.937
Commercial housing	283 (42.0)	104 (40.9)		
Urban village	389 (57.7)	149 (58.7)		
Factory dormitory	2 (0.3)	1 (0.4)		
Age of onset (months)			33.4	< 0.001
0–11	111 (16.5)	77 (30.3)		
12–23	236 (35.0)	97 (38.2)		
24–35	131 (19.4)	42 (16.5)		
36–59	196 (29.1)	38 (15.0)		
Year of onset (years)			102.5	< 0.001
2020	181 (26.9)	36 (14.2)		
2021	290 (43.0)	75 (29.5)		
2022	44 (6.5)	31 (12.2)		
2023	46 (6.8)	75 (29.5)		
2024	113 (16.8)	37 (14.6)		
Month of onset (month)			41.3	< 0.001
1	367 (54.5)	115 (45.3)		
2	148 (22.0)	56 (22.0)		
3	159 (23.6)	83 (32.7)		
RV5 vaccination status ^a^			126.4	< 0.001
No	609 (90.4)	148 (58.3)		
Yes	65 (9.6)	106 (41.7)		
RV5 vaccination doses ^b^			132.4	< 0.001
1 dose	8 (1.2)	4 (1.6)		
2 doses	11 (1.6)	15 (5.9)		
3 doses	46 (6.8)	87 (34.3)		

*Notes*:

^a^ First dose (≥ 15 days before onset).

^b^ All doses (≥ 15 days before onset).

*Abbreviation*: RV5, pentavalent rotavirus.

### 2.7 Sample size for vaccine effectiveness

A precision-based sample size calculation for an unmatched case-control study (assuming vaccine coverage of 40 %, three-dose RV5 efficacy of 80 %, a two-sided significance level [*α*] of 0.05, 80 % power, and a 95 % CI width < 0.2) indicated that a minimum of 229 cases and 229 controls are required to achieve both statistical power and CI precision. The sample size was calculated using the CIs for vaccine efficacy using an unmatched case-control design module in PASS 2021 (version 21.0.3, NCSS LLC, Kaysville, Utah, United States). The calculation formula and detailed results are provided in Supplementary Table S2.

### 2.8 Statistical analysis

A comprehensive database of laboratory-confirmed diarrhea cases in children aged 2–59 months was established using Microsoft Excel 2019. Group comparisons were performed using chi-square tests. Unconditional logistic regression, adjusted for a minimally sufficient set of confounders, was used to calculate adjusted odds ratios (aORs) and 95 % CIs (*α* = 0.05). VE was derived as VE = (1 − aOR) × 100 %.

The final adjustment set included: sex; age at onset (in months, continuous); gestational age (preterm: < 37 weeks; term: ≥ 37 weeks); birth weight (low: < 2500 g; normal: 2500–3999 g; overweight: ≥ 4000 g); neonatal health status (normal, malnourished, obese, or high-risk); feeding modality during 0–6 months (exclusive breast milk, mixed, or formula); year of onset (2020–2024); month of onset (January–March); residential type (commercial housing, urban village, or factory dormitory); residential street; and household registration status (Shenzhen or non-Shenzhen).

Stratified analyses were conducted to evaluate VE by age group and number of vaccine doses. A post hoc subgroup analysis further assessed the modifying effects of gestational age and feeding modality on VE estimates. All analyses were performed using R software (version 4.4.0, R Development Core Team, Vienna, Austria).

### 2.9 Sensitivity analysis

To evaluate the robustness of the VE estimates, sensitivity analyses were conducted using four approaches: (1) omitting adjustment for gender and household registration (*p* > 0.05) to assess the impact of baseline-balanced variables; (2) excluding adjustment for breastfeeding to evaluate VE when breastfeeding may act as a mediator; (3) removing adjustment for residential type to examine potential confounding from socioeconomic proxy variables; and (4) including cases from seasonal transition months (April and December) to assess the impact of non-epidemic-season cases on VE estimates.

### 2.10 Availability of data

Original data containing personal identifiers is securely stored at the Shenzhen Longhua District Maternity and Child Healthcare Hospital. Data access is permitted only in compliance with applicable legal provisions to ensure confidentiality.

## 3 Results

### 3.1 RVGE prevalence

**Fig. 3:**
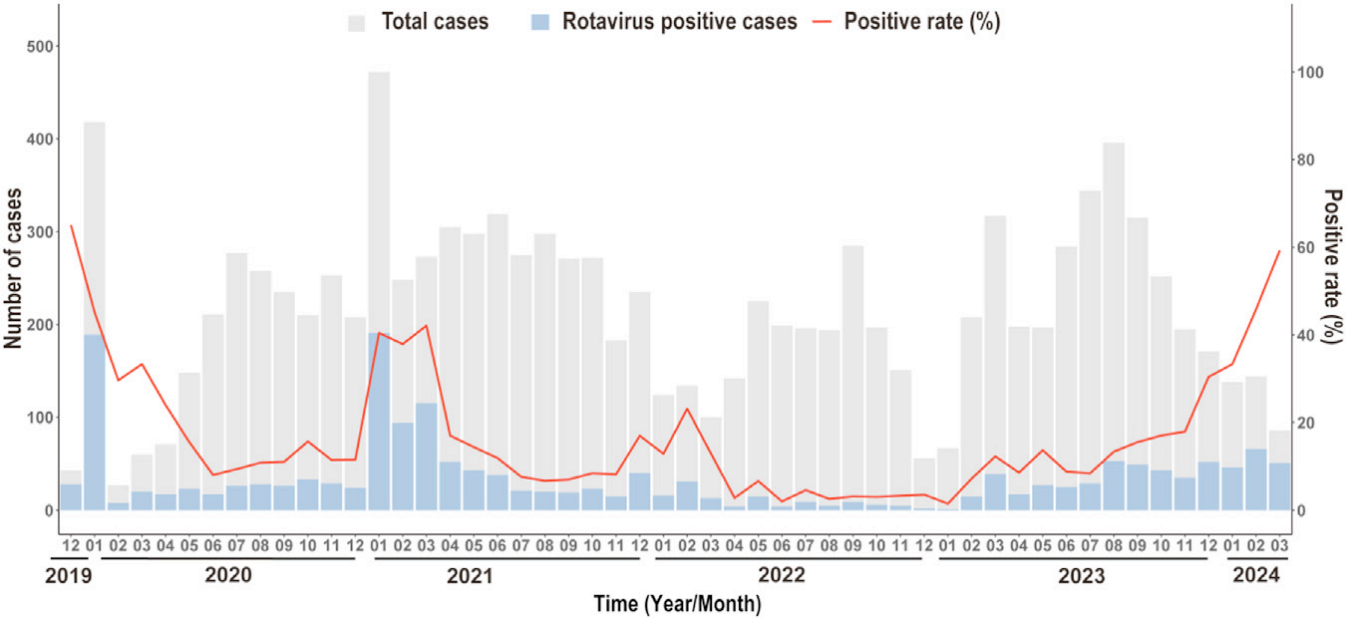
Prevalence and seasonal trends of RVGE from January 2020 to March 2024. *Abbreviation*: RVGE, rotavirus gastroenteritis.

From January 2020 to March 2024, a total of 11,162 cases of AGE were reported in Longhua District, Shenzhen through NDRS. Among these, 1,787 cases (16.0 %) were laboratory-confirmed as rotavirus-positive (RV+). RVGE incidence showed typical seasonal peaks between December and March from January 2020 to April 2022, followed by a progressive decline in epidemic intensity. Notably, no seasonal RVGE peak occurred between April 2022 and January 2023, indicating a disruption in the usual seasonal pattern. Although the RVGE detection rate increased beginning in February 2023, case counts remained stable through March 2024 without the expected January–March epidemic surge. During this period, overall AGE cases continued to decline, in contrast to a significant rise in the RVGE detection rate (Fig. 3).

### 3.2 Demographic characteristics of patients with infectious diarrhea pathogens

After excluding cases lacking laboratory confirmation (*n* = 8,457), those occurring outside the epidemic season (*n* = 1,408), cases with vaccination ≤ 14 days prior to diagnosis (*n* = 14), LLR-vaccinated cases (*n* = 297), and cases with missing vaccination records or key information (*n* = 79), the final analytical amount comprised 928 laboratory-confirmed RVGE cases that met all inclusion criteria for vaccine effectiveness assessment. The RV-positive group included 674 children (72.6 %), of whom 65 (9.6 %) were vaccinated with RV5. Among the 254 RV-negative controls (27.4 %), 106 (41.7 %) were vaccinated. Significant differences were observed between cases and controls regarding residential area, neonatal health status, age at onset, year of onset, and month of onset (*p* < 0.05).

### 3.3 Vaccine effectiveness against RVGE

**Fig. 4:**
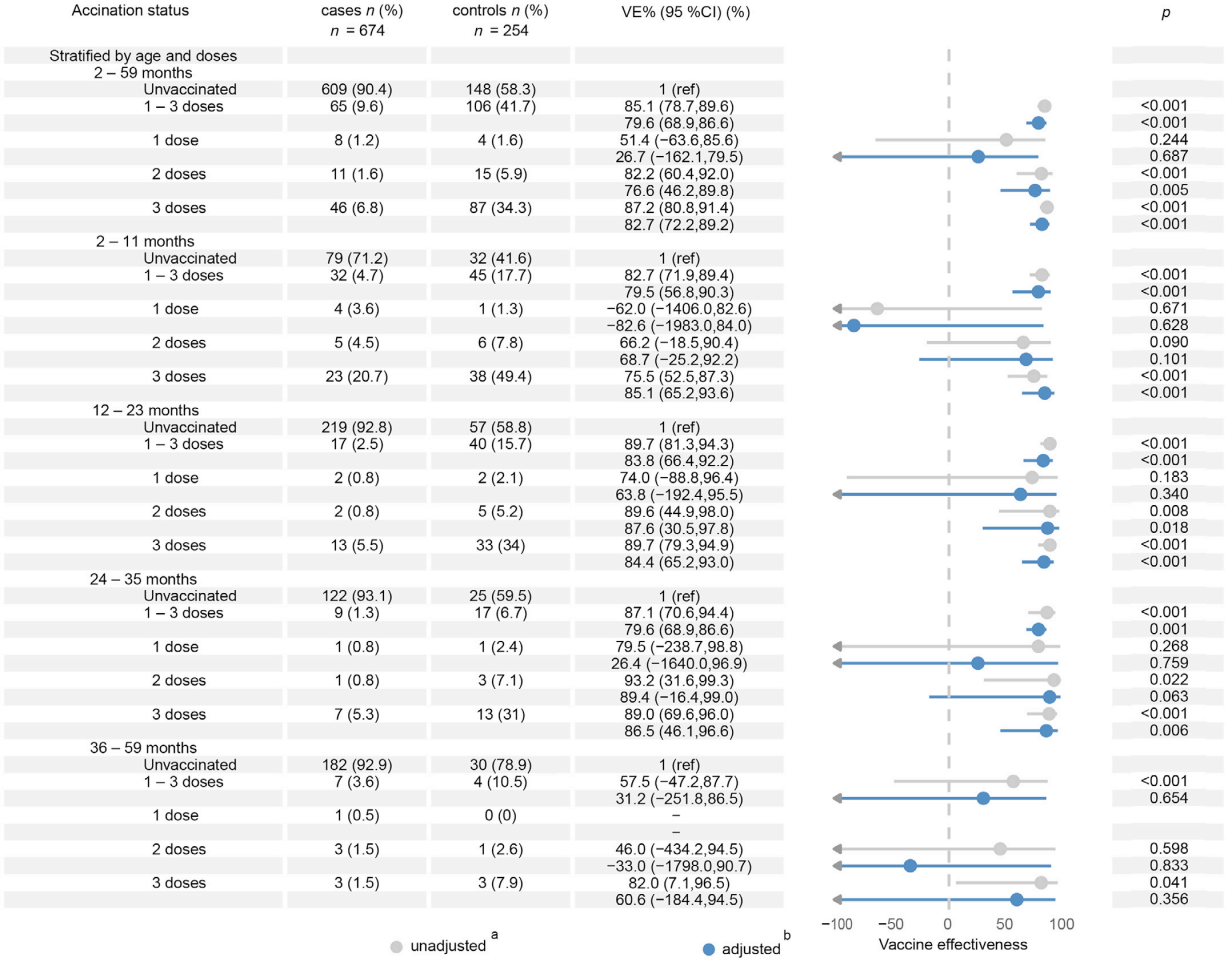
Subgroup analysis of RV5 effectiveness by age and number of vaccine doses. *Notes*: ^a^ Health-seeking behavior was adjusted for in the test negative design. ^b^ The adjustment set comprised: Sex; Age at onset (months; continuous); Gestational age (preterm: < 37 weeks; term: ≥ 37 weeks); Birth weight (low birth weight: < 2500 g; normal weight: 2500–3999 g; overweight: ≥ 4000 g); Neonatal health status (normal/malnourished/obese/high-risk); Feeding modality (0–6 months: exclusive breast milk/mixed/formula); Onset year (2020–2024); Onset month (January–March); Residential type (commercial housing/urban village/factory dormitory); Residential street; Household registration (Shenzhen/non-Shenzhen). *Abbreviations*: VE, vaccine effectiveness; RV5, pentavalent rotavirus.

During RV epidemic seasons, RV5 demonstrated significant dose-dependent protection against RVGE in children under 5 years of age. The adjusted VE was 79.6 % (95 % CI: 68.9 %–86.6 %) for ≥ 1 dose, 76.6 % (95 % CI: 46.2 %–89.8 %) for 2 doses, and 82.7 % (95 % CI: 72.2 %–89.2 %) for 3 doses (all *p* < 0.05) (Fig. 4).

Age-stratified VE: Three doses of RV5 provided substantial effectiveness across younger age groups: 85.1 % (95 % CI: 65.2 %–93.6 %) in children aged 2–11 months, 84.4 % (95 % CI: 65.2 %–93.0 %) in those aged 12–23 months, and 86.5 % (95 % CI: 46.1 %–96.6 %) in the 24–35 month group. Two doses achieved 87.6 % VE (95 % CI: 30.5 %–97.8 %) in children aged 12–23 months. However, two-dose VE was not significant (*p* > 0.05) in children aged 2–11 months, 24–35 months, or 36–59 months. A single dose did not provide significant effectiveness in any age group, including children aged 36–59 months.

**Fig. 5:**
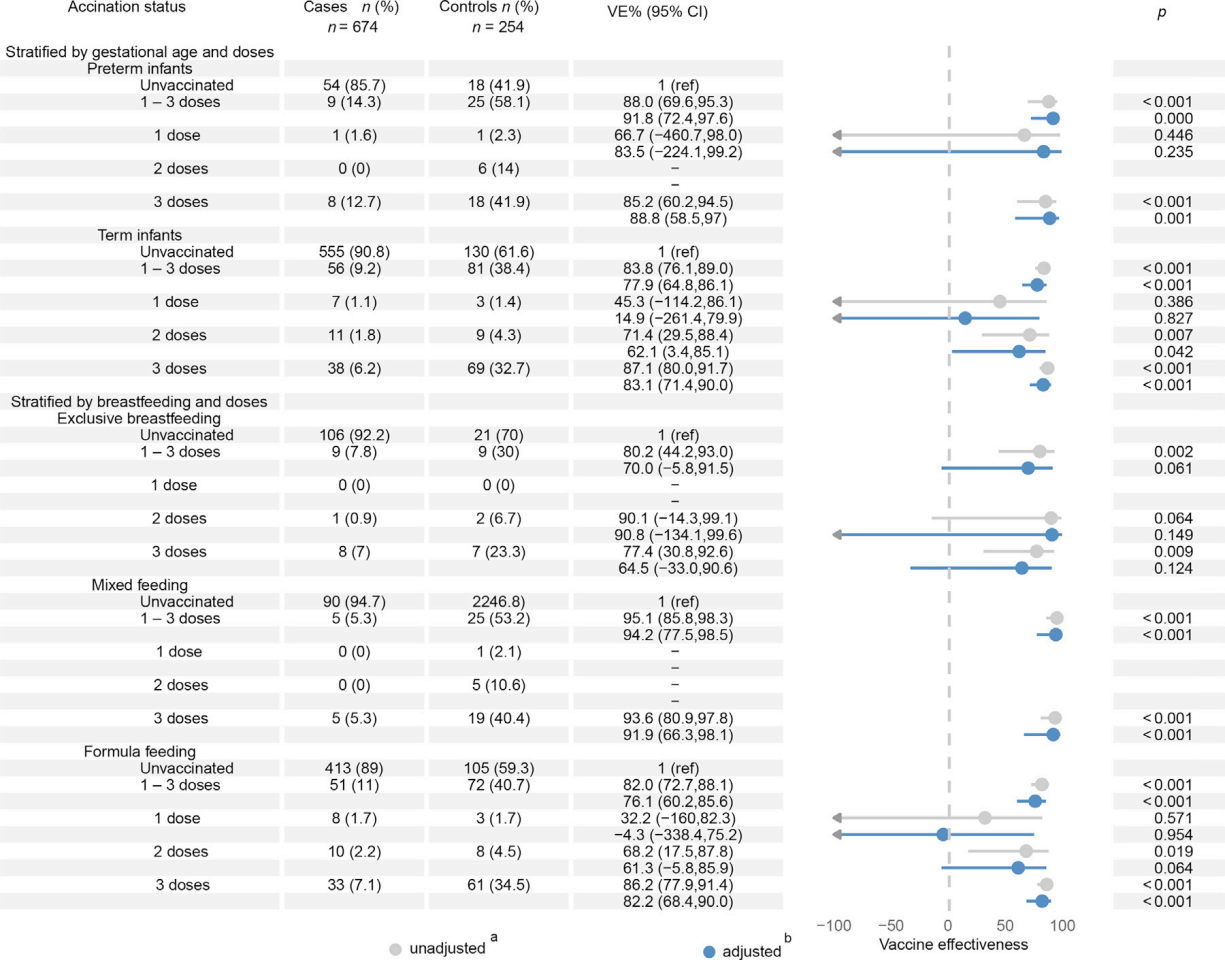
Analysis of RV5 effectiveness in non-prespecified exploratory subgroups (gestational age, breastfeeding status, and number of vaccine doses). *Notes*: ^a^ Health-seeking behavior was adjusted for in the test negative design. ^b^ The adjustment set comprised: Sex; Age at onset (months; continuous); Gestational age (preterm: < 37 weeks; term: ≥ 37 weeks); Birth weight (low birth weight: < 2500 g; normal weight: 2500–3999 g; overweight: ≥ 4000 g); Neonatal health status (normal/malnourished/obese/high-risk); Feeding modality (0–6 months: exclusive breast milk/mixed/formula); Onset year (2020–2024); Onset month (January–March); Residential type (commercial housing/urban village/factory dormitory); Residential street; Household registration (Shenzhen/non-Shenzhen). *Abbreviations*: VE, vaccine effectiveness; RV5, pentavalent rotavirus.

Subgroup analysis (Fig. 5): Preterm infants exhibited significantly higher VE (91.8 %; 95 % CI: 72.4 %–97.6 %) than term infants (77.9 %; 95 % CI: 64.8 %–86.1 %).

Sensitivity analyses (Supplementary Table S3): Excluding adjustments for sex and residential type slightly reduced VE in the 2–11-month age group but did not significantly affect other age groups. VE estimates remained stable across alternative adjustment models. The inclusion of transitional epidemic months (April and December) reduced both overall and age-stratified VE estimates.

## 4 Discussion

Leveraging comprehensive data from the NDRS, this study outlines the latest epidemiological characteristics of AGE in Longhua District, Shenzhen demonstrates the real-world VE of RV5, and highlights the potential application of the surveillance system in public health research. Methodologically, we employed a test-negative design combined with a DAG-derived minimal sufficient adjustment set. This approach improves the reliability of VE estimates by visually identifying and controlling for confounding biases.

Epidemiological trends indicated that strict COVID-19 interventions (2020–2022) significantly suppressed the transmission of RVGE,^48^ leading to reduced seasonal peaks and detection rates. Following China's shift from Class A to Class B infectious disease management in late 2022,^49^ population mobility resumed alongside widespread SARS-CoV-2 infections. Although RVGE detection increased in 2023, no surge in cases was observed during the January–March 2024 epidemic season, and overall AGE reports continued to decline. This pattern suggests that RV5 vaccination—even at suboptimal coverage—may disrupt transmission chains and confer herd immunity among children aged 2–59 months^50^ (Fig. 3).

Within the data evaluating RV5 effectiveness against RVGE in children aged 2–59 months across epidemic seasons, the case group substantially outnumbered the control group. This imbalance does not affect the internal validity of the test-negative design. A key strength of this design lies in its selection of controls from children presenting with acute gastroenteritis, which effectively controls for confounding due to differential healthcare-seeking behavior, while group assignment is determined solely by laboratory confirmation. As long as both groups are drawn from the same healthcare-seeking population, the methodological premise remains valid regardless of group size. Therefore, the observed enrollment imbalance reflects methodological rigor and accurately represents real-world epidemiology and clinical practice.

During epidemic seasons, RVGE accounted for 72.6 % of laboratory-confirmed cases, a finding consistent with the 74.2 % RV-positive rate reported in Hangzhou, Zhejiang Province, China.^36^ Our RV-negative control group (*n* = 254) provided sufficient statistical power. Three doses of RV5 conferred significant effectiveness against epidemic-season RVGE, with a VE of 82.7 % (95 % CI: 72.2 %–89.2 %). This result aligns with VE estimates from Shanghai (85 %–87.1 %),^9^^,^^10^ Guangdong Province (86.6 %),^11^ the United States (83 %),^51^ and other low-child-mortality settings (86 %).^52^ The two-dose regimen achieved an overall effectiveness of 76.6 % (95 % CI: 46.2 %–89.8 %), and 87.6 % (95 % CI: 30.5 %–97.8 %) among children aged 12–23 months, though limited statistical power reduced significance in other age groups. Single-dose vaccination showed no significant effectiveness, likely due to insufficient immunogenicity rather than sample size limitations. The superiority of multi-dose schedules arises from their ability to simulate natural rotavirus exposure,^53^, ^54^, ^55^ eliciting durable serum IgA and mucosal immunity^56^^,^^57^ that collectively reduce infection risk and disease severity—an outcome not achievable with single-dose immunization (RV5 or LLR).^58^

Age-stratified analyses revealed sustained and significant VE in children aged 2–11 months (85.1 %; 95 % CI: 65.2 %–93.6 %), 12–23 months (84.4 %; 95 % CI: 65.2 %–93.0 %), and 24–35 months (86.5 %; 95 % CI: 46.1 %–96.6 %). Protection waned in children aged ≥ 36 months, potentially due to: (1) ≥ 95 % seropositivity resulting from natural infections,^59^ which may attenuate observable VE against typically milder disease; and (2) age-dependent decay of vaccine-induced immunity.^60^^,^^61^ Importantly, RV5 maintained 85.1 %–86.5 % effectiveness during the high-risk period of 2–35 months—consistent with global data^56^^,^^62^—when the burden of rotavirus disease is greatest. Additionally, among children aged 36–59 months, vaccination primarily occurred during the early implementation phase of RV5, resulting in suboptimal coverage that limited our statistical power to detect protective effects. Nevertheless, the exclusion of RV5 from China's National Immunization Program constrains coverage, thereby limiting herd effects and population-level benefits.^12^^,^^13^

A post hoc analysis suggested higher VE in preterm infants (90.7 %; 95 % CI: 70.2 %–97.1 %), which may be attributable to: (1) reduced interference from maternal antibodies (associated with shorter breastfeeding duration^63^); and (2) increased care-seeking behavior for severe RVGE.^64^ However, this finding requires validation in larger data with severity stratification, given the limited sample size of preterm infants in our study (*n* = 106).

This study has several limitations. First, the high detection threshold of the colloidal gold immunoassay (1 × 10³ copies/g for RV) may increase the risk of false negatives in mild cases and samples with low viral loads, potentially reducing the accuracy of VE estimates during non-epidemic seasons and in mild RVGE presentations. Second, potential misclassification of mild RVGE cases as controls may have downwardly biased VE estimates. Third, reduced statistical power in stratified and subgroup analyses—evidenced by wide CIs (e.g., for two-dose VE in children aged 2–11 and 24–35 months, and for any dose in children aged 36–59 months)—increases the risk of type II errors. Fourth, residual confounding may persist due to the use of simplified socioeconomic proxies (e.g., residence type) and unmeasured factors such as parental education, which could bias VE estimates through indirect causal pathways. Fifth, although existing evidence supports cross-protection against prevalent G8P[8] strains,^8^^,^^11^ the lack of strain-specific genotyping prevents direct assessment of vaccine performance against circulating variants. Finally, the urban sampling frame limits generalizability to rural and low-resource settings, where vaccination uptake is typically lower.

## 5 Conclusion

The RV5 vaccine demonstrates significant and dose-dependent protection against epidemic-season RVGE. A three-dose regimen confers high effectiveness in children under 36 months of age, with preliminary evidence suggesting even greater protection among preterm infants. Although these findings underscore the high real-world effectiveness of RV5 and support its potential inclusion in national immunization programs, the current high cost remains a major barrier to accessibility. Further studies are warranted to establish a comprehensive evidence base regarding both the effectiveness and cost-effectiveness of RV5, as well as domestically produced alternatives such as the LLR3 vaccine, in reducing the burden of RVGE. Such research will be essential for advancing and optimizing rotavirus vaccination strategies.

## Appendix Supplementary materials

Image, application 1

Image, application 2

Image, application 3

Image, application 4
