# Supplementary material for: Pentavalent rotavirus vaccine effectiveness among children in Shenzhen, China: A population-based test-negative design with directed acyclic graphs bias adjustment
Source: Infect Med (Beijing). 2025 Sep 5;4(3):100201. doi: 10.1016/j.imj.2025.100201 (PMC12541608; doi:10.1016/j.imj.2025.100201)
Supplement: Supplementary file 1 [file mmc1.docx]

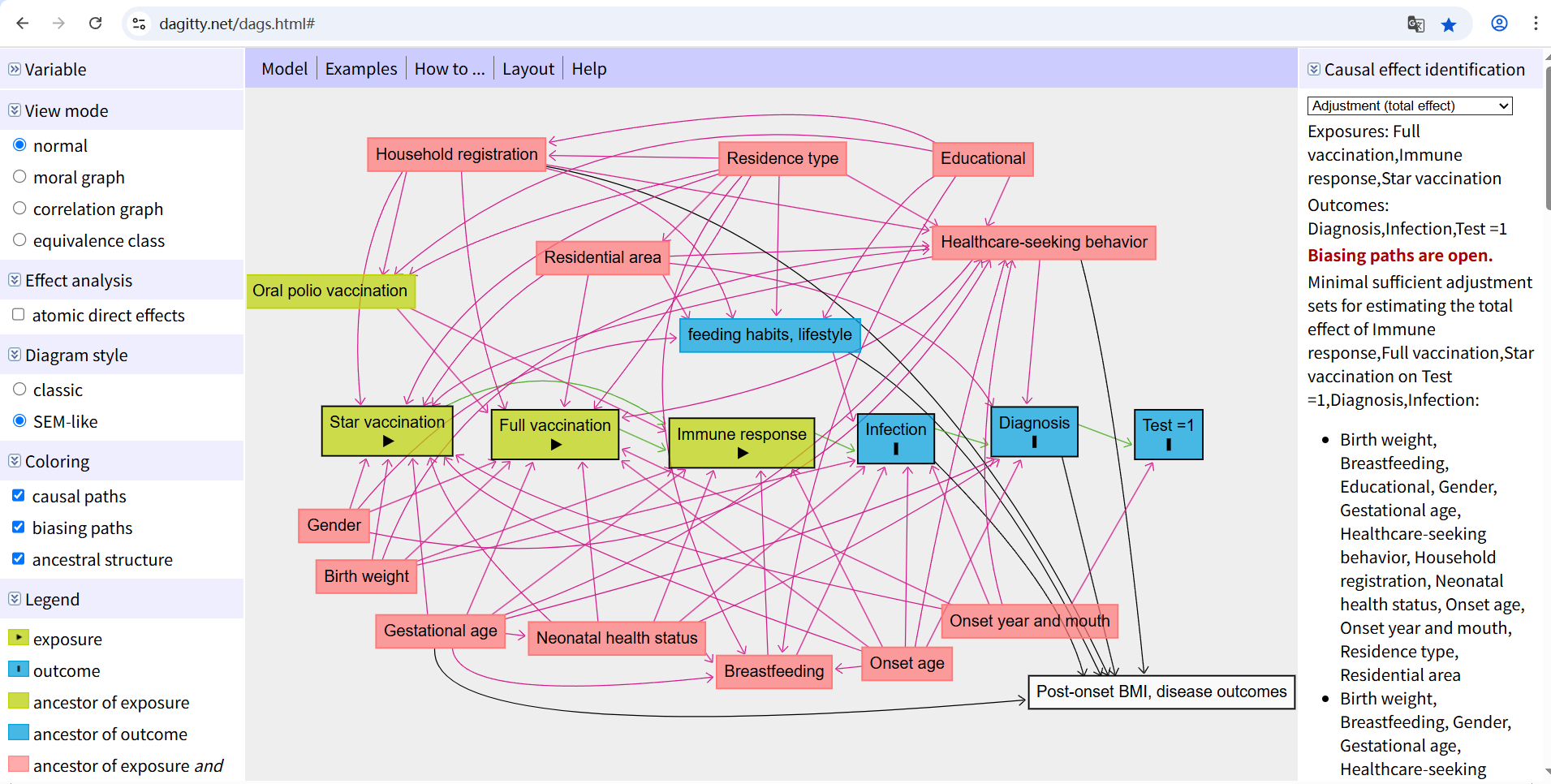


**Fig. S1.** Original detailed directed acyclic graph.

Minimal sufficient adjustment sets for estimating the total effect of Immune response, Full vaccination, Star vaccination on Test =1, Diagnosis, Infection:

- Birth weight, Breastfeeding, Educational, Gender, Gestational age, Healthcare-seeking behavior, Household registration, Neonatal health status, Onset age, Onset year and mouth, Residence type, Residential area
- Birth weight, Breastfeeding, Gender, Gestational age, Healthcare-seeking behavior, Household registration, Neonatal health status, Onset age, Onset year and mouth, Oral polio vaccination, Residence type, Residential area
- Birth weight, Breastfeeding, Gestational age, Healthcare-seeking behavior, Neonatal health status, Onset age, Onset year and mouth, Residential area, feeding habits, lifestyle

### Model code

dag {

bb="0,0,1,1"

"Birth weight" [pos="0.114,0.695"]

"Full vaccination" [exposure,pos="0.294,0.481"]

"Gestational age" [pos="0.185,0.772"]

"Healthcare-seeking behavior" [pos="0.760,0.220"]

"Household registration" [pos="0.200,0.095"]

"Immune response" [exposure,pos="0.472,0.493"]

"Neonatal health status" [pos="0.353,0.782"]

"Onset age" [pos="0.630,0.818"]

"Onset year and mouth" [pos="0.747,0.758"]

"Oral polio vaccination" [pos="0.079,0.290"]

"Post-onset BMI, disease outcomes" [pos="0.872,0.859"]

"Residence type" [pos="0.511,0.101"]

"Residential area" [pos="0.339,0.242"]

"Star vaccination" [exposure,pos="0.134,0.476"]

"Test =1" [outcome,pos="0.879,0.481"]

"feeding habits, lifestyle" [pos="0.499,0.352"]

Breastfeeding [pos="0.503,0.830"]

Diagnosis [outcome,pos="0.751,0.477"]

Educational [pos="0.702,0.102"]

Gender [pos="0.083,0.622"]

Infection [outcome,pos="0.619,0.487"]

"Birth weight" -> "Full vaccination"

"Birth weight" -> "Healthcare-seeking behavior" [pos="0.222,0.281"]

"Birth weight" -> "Immune response"

"Birth weight" -> "Star vaccination"

"Birth weight" -> Infection

"Full vaccination" -> "Immune response"

"Gestational age" -> "Full vaccination"

"Gestational age" -> "Healthcare-seeking behavior" [pos="0.544,0.589"]

"Gestational age" -> "Immune response"

"Gestational age" -> "Neonatal health status"

"Gestational age" -> "Post-onset BMI, disease outcomes" [pos="0.172,0.941"]

"Gestational age" -> "Star vaccination"

"Gestational age" -> Breastfeeding [pos="0.201,0.876"]

"Gestational age" -> Diagnosis [pos="0.560,0.634"]

"Healthcare-seeking behavior" -> "Full vaccination" [pos="0.606,0.412"]

"Healthcare-seeking behavior" -> "Post-onset BMI, disease outcomes" [pos="0.828,0.429"]

"Healthcare-seeking behavior" -> "Star vaccination" [pos="0.228,0.357"]

"Healthcare-seeking behavior" -> Diagnosis

"Household registration" -> "Full vaccination" [pos="0.211,0.324"]

"Household registration" -> "Healthcare-seeking behavior"

"Household registration" -> "Oral polio vaccination"

"Household registration" -> "Post-onset BMI, disease outcomes" [pos="0.622,0.205"]

"Household registration" -> "Star vaccination" [pos="0.095,0.246"]

"Household registration" -> "feeding habits, lifestyle" [pos="0.432,0.166"]

"Immune response" -> Infection

"Neonatal health status" -> "Full vaccination"

"Neonatal health status" -> "Immune response"

"Neonatal health status" -> "Star vaccination" [pos="0.194,0.625"]

"Neonatal health status" -> Breastfeeding

"Neonatal health status" -> Diagnosis [pos="0.584,0.656"]

"Neonatal health status" -> Infection

"Onset age" -> "Full vaccination"

"Onset age" -> "Healthcare-seeking behavior" [pos="0.671,0.510"]

"Onset age" -> "Immune response"

"Onset age" -> "Star vaccination" [pos="0.231,0.618"]

"Onset age" -> Breastfeeding

"Onset age" -> Diagnosis

"Onset age" -> Infection

"Onset year and mouth" -> "Full vaccination"

"Onset year and mouth" -> "Healthcare-seeking behavior" [pos="0.683,0.543"]

"Onset year and mouth" -> "Star vaccination" [pos="0.311,0.634"]

"Onset year and mouth" -> "Test =1"

"Onset year and mouth" -> Infection

"Oral polio vaccination" -> "Full vaccination"

"Oral polio vaccination" -> "Immune response"

"Residence type" -> "Full vaccination" [pos="0.420,0.291"]

"Residence type" -> "Healthcare-seeking behavior"

"Residence type" -> "Household registration"

"Residence type" -> "Oral polio vaccination" [pos="0.239,0.190"]

"Residence type" -> "Residential area"

"Residence type" -> "Star vaccination" [pos="0.206,0.239"]

"Residence type" -> "feeding habits, lifestyle"

"Residence type" -> Breastfeeding [pos="0.319,0.383"]

"Residential area" -> "Full vaccination"

"Residential area" -> "Healthcare-seeking behavior"

"Residential area" -> "Star vaccination" [pos="0.219,0.330"]

"Residential area" -> "feeding habits, lifestyle"

"Residential area" -> Diagnosis [pos="0.647,0.288"]

"Star vaccination" -> "Immune response" [pos="0.297,0.370"]

"feeding habits, lifestyle" -> "Post-onset BMI, disease outcomes" [pos="0.705,0.491"]

"feeding habits, lifestyle" -> Infection

Breastfeeding -> "Immune response"

Breastfeeding -> Infection

Diagnosis -> "Post-onset BMI, disease outcomes"

Diagnosis -> "Test =1"

Educational -> "Healthcare-seeking behavior"

Educational -> "Household registration" [pos="0.561,-0.001"]

Educational -> "Oral polio vaccination" [pos="0.349,-0.001"]

Educational -> "feeding habits, lifestyle" [pos="0.613,0.163"]

Educational -> Breastfeeding [pos="0.540,0.424"]

Gender -> "Full vaccination"

Gender -> "Healthcare-seeking behavior" [pos="0.508,0.751"]

Gender -> "Star vaccination"

Gender -> "feeding habits, lifestyle" [pos="0.234,0.366"]

Infection -> "Post-onset BMI, disease outcomes" [pos="0.777,0.712"]

Infection -> Diagnosis

}
