# Supplementary material for: Pentavalent rotavirus vaccine effectiveness among children in Shenzhen, China: A population-based test-negative design with directed acyclic graphs bias adjustment
Source: Infect Med (Beijing). 2025 Sep 5;4(3):100201. doi: 10.1016/j.imj.2025.100201 (PMC12541608; doi:10.1016/j.imj.2025.100201)
Supplement: Supplementary file 3 [file mmc3.zip › Table S2.rtf]

PASS 2021, v21.0.3	2025/8/2 18:58:03      1

Supplementary Table S2
Confidence intervals for vaccine efficacy using an unmatched case-control design.

Numeric Results ────────────────────────────────────────────────────────────
Interval Type:	Two-Sided
Confidence Interval Method:	Mantel-Haenszel

										Lower	Upper
				Target	Actual	Relative	Vaccine		Conf	Conf
Conf				C.I.	C.I.	C.I.	── Prevalence ──	Vaccine	Limit	Limit
Level	Case	Control		Width	Width	Width	Case	Control	Efficacy	of VE	of VE
1 - α	N1	N2	N	Wᴛ	Wᴀ	RW	P1	P2	VE	LCL	UCL
0.95	229	229	458	0.2	0.19997	0.24996	0.11765	0.4	0.8	0.67642	0.87638

References
O'Neill, Robert T. 1988. 'On Sample Sizes to Estimate the Protective Efficacy of a Vaccine'. Statistics in Medicine,
   Volume 7, Pages 1279-1288.
Farrington, C. P. and Manning, G. 1990. 'Test Statistics and Sample Size Formulae for Comparative Binomial
   Trials with Null Hypothesis of Non-Zero Risk Difference or Non-Unity Relative Risk.' Statistics in Medicine, Vol.
   9, pages 1447-1454.
Fleiss, J. L., Levin, B., Paik, M.C. 2003. Statistical Methods for Rates and Proportions. Third Edition. John Wiley &
   Sons. New York.
Miettinen, O.S. and Nurminen, M. 1985. 'Comparative analysis of two rates.' Statistics in Medicine 4: 213-226.
Robbins, Breslow, and Greenland. 1986. 'Estimators of the Mantel-Haenszel Variance Consistent in both Sparse
   Data and Large-Strata Limiting Models.' Biometrics, Volume 42, 311-323.
Sahai, H. and Khurshid, A. 1995. Statistics in Epidemiology. CRC Press. Boca Raton, Florida.
Schlesselman, James. 1982. Case-Control Studies: Design, Conduct, Analysis. Oxford University Press. New
   York.

Report Definitions
Confidence level is the proportion of confidence intervals (constructed with this same confidence level, sample
   size, etc.) that would contain the true value of VE.
N1 is the number of subjects sampled from the population of cases.
N2 is the number of subjects sampled from the population of controls.
N is the total sample size, N1 + N2.
Wᴛ is the target width of the confidence interval of VE.
Wᴀ is the actual width of the confidence interval of VE that was computed by the procedure.
RW is the relative width of the confidence interval. RW = Width / VE.
P1 is the probability of having been vaccinated (vaccine prevalence) in the case group.
P2 is the probability of having been vaccinated (vaccine prevalence) in the control group.
VE is the index of vaccine efficacy. It represents the proportion of cases of disease prevented by the vaccine. It is
   calculated using VE = 1 - OR, where OR is the odds ratio of the prevalences of cases to controls.
LCL is the lower confidence limit of VE.
UCL is the upper confidence limit of VE.


Summary Statements ─────────────────────────────────────────────────────────
Sample sizes of 229 from the cases and 229 from the controls achieve a width of 0.19997 using a two-sided
confidence interval of VE based on the Mantel-Haenszel method. The vaccine efficacy (VE) is assumed to be 0.8.
The confidence level of the interval is 0.95. The prevalence of vaccine exposure among the cases is 0.11765.
The prevalence of vaccine exposure among the controls is 0.4. 


PASS 2021, v21.0.3	2025/8/2 18:58:03      2

Confidence Intervals for Vaccine Efficacy using an Unmatched Case-Control Design

Dropout-Inflated Sample Size ────────────────────────────────────────────────────

						Dropout-Inflated		Expected
						Enrollment		Number of
		─── Sample Size ───		─── Sample Size ───		─── Dropouts ───
Dropout Rate		N1	N2	N		N1'	N2'	N'		D1	D2	D
20%		229	229	458		287	287	574		58	58	116

Definitions
Dropout Rate (DR) is the percentage of subjects (or items) that are expected to be lost at random during the
   course of the study and for whom no response data will be collected (i.e., will be treated as "missing").
N1, N2, and N are the evaluable sample sizes at which power is computed. If N1 and N2 subjects are evaluated
   out of the N1' and N2' subjects that are enrolled in the study, the design will achieve the stated power.
N1', N2', and N' are the number of subjects that should be enrolled in the study in order to end up with N1, N2,
   and N evaluable subjects, based on the assumed dropout rate. After solving for N1 and N2, N1' and N2' are
   calculated by inflating N1 and N2 using the formulas N1' = N1 / (1 - DR) and N2' = N2 / (1 - DR), with N1' and
   N2' always rounded up. (See Julious, S.A. (2010) pages 52-53, or Chow, S.C., Shao, J., Wang, H., and
   Lokhnygina, Y. (2018) pages 32-33.)
D1, D2, and D are the expected number of dropouts. D1 = N1' - N1, D2 = N2' - N2, and D = D1 + D2.


Procedure Input Settings ──────────────────────────────────────────────────────

Autosaved Template File
C:\Users\ÁÖ×Ó°²\Documents\PASS 2021\Procedure Templates\Autosave\Confidence Intervals for Vaccine Efficacy using an Unmatched Case-Control Design - Autosaved 2025_8_2-18_58_3.t707

Design Tab
Solve For:	Sample Size
Method:	Mantel-Haenszel
Interval Type:	Two-Sided
Confidence Level (1 - α):	0.95
Group Allocation:	Equal (N1 = N2)
Precision Input Type:	Absolute
W (Confidence Interval Width):	0.2
Vaccine Efficacy Input Type:	Enter VE and P2
VE (Vaccine Efficacy):	0.8
P2 (Prevalence of Vaccine in Controls):	0.4

Options Tab
Maximum N1 Before Search Termination:	Default
